# An improved Kepler optimization algorithm for module parameter identification supporting PV power estimation

**DOI:** 10.1016/j.heliyon.2024.e39902

**Published:** 2024-10-30

**Authors:** Ghareeb Moustafa, Hashim Alnami, Ahmed R. Ginidi, Abdullah M. Shaheen

**Affiliations:** a Department of Electrical and Electronic Engineering, College of Engineering and Computer Science, Jazan University, P.O. Box114, Jazan, 45142, Saudi Arabia; b Department of Electrical Engineering, Faculty of Engineering, Suez University, P.O. Box: 43221, Suez, Egypt

**Keywords:** Kepler optimization algorithm, Local Escaping Operator, PV parameters extraction, Practical solar modules

## Abstract

Identification of photovoltaic (PV) module characteristics in solar systems is a vital task, nowadays, for optimal PV power estimation. In this paper, this challenge task has been studied using a novel advanced Kepler optimization algorithm (KOA). The standard version of KOA is adopted and assessed for getting the nine parameters of the PV triple diode model (3DM) considering three different practical PV modules. Kepler's principles of planetary motion are used by KOA to forecast the location and velocity of planets at any particular moment. However, the success rate of the KOA is not compatible, and its efficiency needs to be enhanced. As a result, an Improved KOA (IKOA) is created by incorporating an advanced mechanism of Local Escaping Operator (LEO), resulting in improved process of searching with evading local optima. This mechanism means that the exploitation approach will activate with around half of the solutions for every iteration starting at the initial phase of the iteration journey. The suggested IKOA besides the standard KOA are developed for predicting PV parameters for three distinct PV modules which are Photowatt PWP201, R.T.C France and STM6-40/36. The results corresponding to the latest algorithms are also compared with the proposed IKOA about different published works. The simulation findings reveal that the suggested IKOA exhibits notable average improvement rates for the three modules of 62.27 %, 55.1 %, and 32.12 %, respectively. Furthermore, the suggested IKOA asserts significant superiority and robustness over previously reported results.

## 1 Introduction

Nonconventional sources of energy, including solar power, are gaining a lot of attention due to rising environmental concerns and rising energy demands. Lightweight solar energy may be easily converted into electrical energy with the use of photovoltaic (PV) cells. Green energy conversion, low maintenance costs, and the absenteeism or occurrence of revolving apparatuses are only a few of the major advantages that come with the realization of PV sources [1,2]. The need for numerous resources, especially energy, is continually rising due to the world's rapid development. Burning greet amount of fossil fuels will result in environmental degradation, fossil fuels to run out, and cause climate change [[3], [4], [5]]. Thus, switching from fossil fuels to renewable energy has developed as the favoured response for these issues. Geothermal, Wind, solar energy, and biomass [6], are all types of renewable energies. Solar energy is regarded to have the most potential of any other kind of energy because of its purity, absence of pollution, long-term use, and large reserves [7]. Studying photovoltaics is crucial since these systems are the best complement to other conventional and renewable energies in hybrid energy systems, which are thought to be the most common in the world due to their stability and dependability in generating energy from separate systems, such as: PV/Wind/Battery [8], PV/Grid [9], Fuel cell [10], PV/Thermal [11], PV/Wind [12], PV [13], PV/Wind/Diesel/Bioenergy [14], PV energy forecasting [15] and predictive control for microgrids [16].

Precise model parameters are required in order to fix the characteristic curves of PV modules or cells for quality control, tracking the maximum power point, and performance assessment of solar systems under various operating situations. As a result, it has always been crucial to collect the essential model parameters with high precision and dependability, and in recent years, this subject has entertained myriads of attention [17]. Hence, numerous researchers are enhancing the parameters of PV models and utilizing a range of design approaches that fall under the categories of deterministic, analytical, and metaheuristic. To determine the values of the unidentified parameters, analytical processes use a series of mathematical equations. However, the results derived from these processes are not precise since they make assumptions in advance [18,19]. In Ref. [20], an artifact heuristic method has been combined with Newton Raphson approach for parameters extraction of different standard PV cells design. In order to operate the solar PV arrays at their greatest power generation, a variety of maximum power point tracking (MPPT) techniques have been established such as artificial neural network, fuzzy logic control, perturb and observe and ripple correction control [21,22]. In previous studies [23,24], a PSO with chaotic characteristics was employed to develop an advanced controller, incorporating power system stabilizer (PSS) with a static VAR compensator. This controller was designed to effectively mitigate power system inter-area fluctuations and significantly improve the dynamic stability of the multi-machine power system. Additionally, in another study [25], gravitational searching optimizer was utilized to design a thyristor-controlled series capacitor, in conjunction with a PSS.

In [26], An advanced optimizer inspired by Ali Baba and the Forty Thieves (AFT), in conjunction with the Newton Rapson (NR) method, was employed to estimate the parameters of solar PV systems, testing on STP6-120/36, STM6-40/36, and PWP201 systems. In this study, the AFT algorithm has shown superior performance over existing techniques through experimental results. However, the AFT algorithm was applied with higher computational burden utilizing and a maximum iteration of 700 and number of thieves of 700 which characterized higher number of function evaluations of 490,000. In Ref. [27], the parameter estimation of solar PV models has been described as a popular benchmark function to assess the performance and effectiveness of the metaheuristic optimizers considering five design variables. This study was shortened only for the simplest PV modelling of 1DM equivalent circuit. In Ref. [28], a newly developed meta-heuristic algorithm of bonobo optimizer has been performed and combined with the Newton-Raphson approach for parameter estimation of solar PV utilizing the 1DM, 2DM and 3DM. In Ref. [29], a hybridized evolutionary Flower Grey Differential (HFGD) algorithm has been presented which was based on the hybridization of GWO, DE, and the Flower Pollination Algorithm (FPA) algorithms. The presented technique has been applied for PV parameter extraction for Amorphous Silicon and PVM 752 GaAs Thin-Film. However, the utilized equivalent circuits were represented by 1DM and 2DM only. Several photovoltaic solar module models, including the one-diode model (1DM) [30,31], two-diode model (2DM) [32], and triple-diode model (3DM) [33], have been presented in order to efficiently use solar energy. The significant model parameter values determine the precision of these photovoltaic module models. The parameters, though, are typically unidentified because they are not listed on the data sheet illustrated by the manufacturer and change depending on the working environment (for instance, as a device ages and fails) [34,35].

Myriads of drawbacks are associated with the deterministic methods when utilized for solar PV models parameter approximation which can be summarized as follows they are sensitive to initial conditions, get trapped in local optima, are computationally intensive, converge to a poor solution, are not robust to outliers and noise, and cannot handle nonlinear and complex models, multi-objective optimization problems, and non-differentiable functions [36]. To avoid aforementioned drawbacks, metaheuristic algorithms have been developed in order not to stuck in local minima. These metaheuristic approaches produce consistent findings for estimating the PV model parameters when compared to other methodologies [37]. In Ref. [38], the Nelder-Mead simplex method has been emerged with the Laplace's cross search mechanism (NMSELC) and employed on the solar cells and PV modules.

I. In recent years, significant investigation has been conducted into the topic of study. Numerous metaheuristic and analytical techniques were manifested by investigators to assess the properties of the solar module or cell (An outline of several of these investigates are provided in the appendix). In spite of the researchers are developing and improving meta-heuristic approaches to establish the parameters of PV models, some algorithms have not successfully struck a compromise between reliability and accuracy while keeping a reasonable computing time. Metaheuristic algorithms need to perform better, thus new approaches must be applied to create effective solutions to establish the PV parameters. Recently, Mohamed Abdel-Basset et al. [39] introduced a unique physics-based metaheuristic method named Kepler optimization algorithm (KOA), influenced by Kepler's equations of planetary motion. Based on Kepler's formulas regarding the prediction of the planets location and velocity at a particular moment, KOA is a distinctive metaheuristic version in the category of physics-based inspiration [39]. KOA is a unique metaheuristic version in the field of physics-based inspiration, that utilizes Kepler's calculations for predicting the planet's placement and velocity at a specific moment [39]. Within KOA, every planet functions as a potential solution, whose position is arbitrarily changed in respect to the Sun, which stands for the ideal candidate solution, throughout the optimization process. KOA strikes a balance among exploration and exploitation by modeling potential solutions as planets whose positions are arbitrarily changed throughout the optimization. Besides, KOA's consideration of practical constraints was successfully demonstrated for economic power and heat dispatch optimization [40] ensuring its relevance and applicability to real-world scenarios. Moreover, A modification using the Fractional Order has been developed for the KOA in Ref. [41], however, this modification has been applied on the 1DM and 2DM only. This approach is assessed in this article to identify the PV design. The PV triple diode model (3DM)'s nine parameters are extracted using the KOA while considering three different real-world PV modules. Nevertheless, the KOA's success rate is incompatible, and its effectiveness must be improved. To improve the process of searching while avoiding local optima, an Improved KOA (IKOA) is produced by including an upgraded Local Escaping Operator (LEO) mechanism. Based on this approach, the activation of the exploitation technique will commence at the beginning of the iteration trip with around 50 % of the answers for each iteration.

In brief, the essential outcomes of this study are as follows.

⁃ An upgraded IKOA based on LEO mechanism is presented.
⁃ To increase the precision in identifying the PV parameters regarding the 3DM representation, the proposed IKOA shows great enhancement versus the standard KOA.
⁃ From the viewpoint of numerous statistical findings, the proposed IKOA is a more competitive method for determining PV model parameters compared to other recently published techniques.

The remaining sections of the document are ordered as follows: the mathematical representation of the 3DM design related to PV cells and modules are offered in Section 2. Additionally, section 3 delivers details about the KOA and the proposed IKOA approaches, including a mathematical model and a flow chart. The experimental findings and analyses are exhibited in Section 4. The work's conclusion is summarized in section 5.

## 2 Representations of PV modules: electrical circuit and mathematical model

PV model parameter extraction remains a difficult issue due to the multi-modality, multi-variability, and nonlinearity characteristics. Separated comparable circuits have been constructed to emphasize the I-V characteristics of PV modules. The 3DM representation is described below [42] and it is considered as the most comprehensive one which depends on extracting nine parameters. The Shockley-diode equivalent circuits have been a popular approximation of PV cells in recent decades.

### 2.1 PV cell modelling using 3DM design

**Fig. 1:**
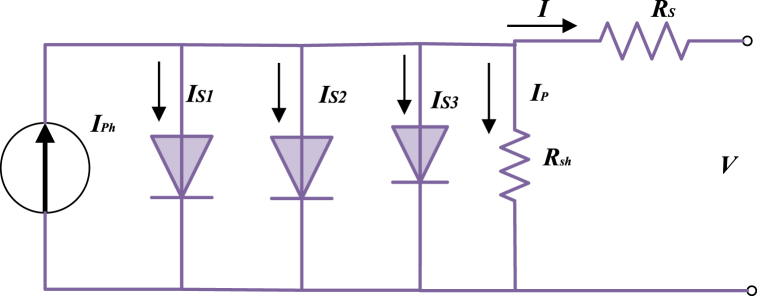
Electrical circuit design of 3DM.

**Fig. 2:**
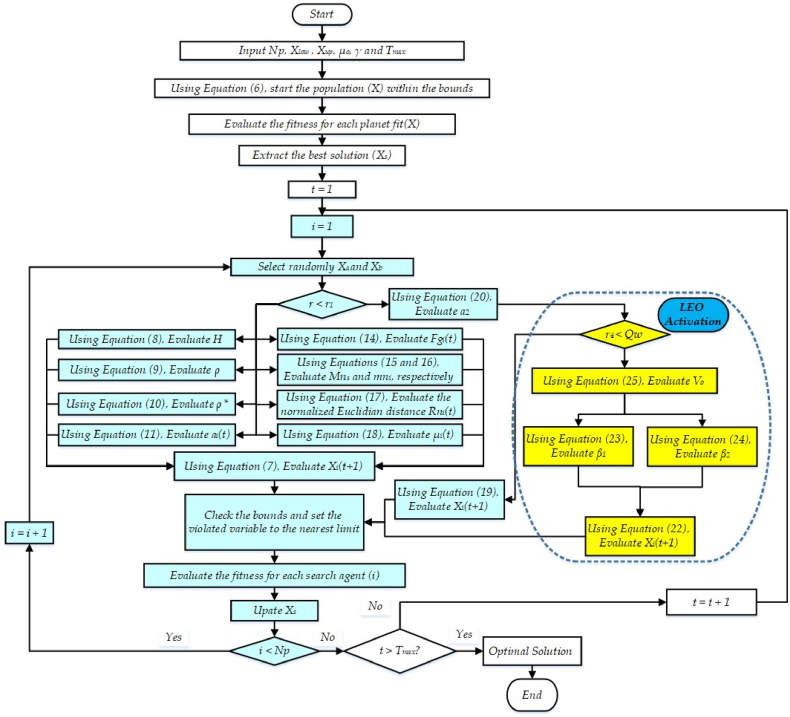
Proposed IKOA flowchart.

Modeling has evolved into an indispensable tool for investigating the dynamic relationships among different elements of a PV system. The 3DM design is generally employed to illustrate the properties of solar cells. Fig. 1 depicts the 3DM design equivalent circuit, in which the PV panel is highlighted as a current source and is connected in parallel with three diodes. The fundamental elements of the 3DM design contained in PV cells are three diodes, two resistors, and a current source, as can be illustrated in Fig. 2. Equation (1) demonstrates the load current equation for the 3DM design [43]:

(1) $$I=I_{Ph}-I_{S1}\left[e^{\left(\frac{V+IR_{S}}{V_{th}}\right)/\eta_{1}\;}-1\right]-I_{S2}\left[e^{\left(\frac{V+IR_{S}}{V_{th}}\right)/\eta_{2}}-1\right]-I_{S3}\left[e^{\left(\frac{V+IR_{S}}{V_{th}}\right)/\eta_{3}}-1\right]-\left(V+IR_{S}\right)/R_{sh}$$

where *I* indicates the output current of the module; *I*_*Ph*_ displays the incident module photocurrent; *I*_*S*1_, *I*_*S*2_ and *I*_*S*3_ express the reverse saturation currents of the three diodes, respectively; *V*_*th*_ characterizes the modules' thermal voltage defined in equation (2); *V* symbolizes the terminal voltage; *R*_*S*_ and *R*_*Sh*_ illustrate the series and shunt resistances that are lumped indicating the losses in the module; *η*_1_, *η*_2_ and *η*_3_ denote the ideality factor of D1, D2 and D3, respectively [44,45].

(2) $$V_{th}=K_{B}\times \frac{T}{q_{c}}$$

where *K*_*B*_ characterizes Boltzmann's coefficient and *qc* characterizes electron charge, while *T* demonstrates the absolute temperature.

### 2.2 Representation of PV modules via 3DM design

The 3DM design equation may be denoted by a PV module with *N*_*sh*_ cells connecting in parallel and *N*_*s*_ cells coupled in series. Thus, equation (1) has been updated and adjusted as depicted in Equation (3) [46]:

(3) $$I=N_{p}\left(I_{ph}-I_{S1}\left[e^{\frac{V+IR_{S}\left(N_{s}/N_{sh}\right)}{\eta_{1}\;N_{s}V_{th}}}-1\right]-I_{S2}\left[e^{\frac{V+IR_{S}\left(N_{s}/N_{sh}\right)}{\eta_{2}\;N_{s}V_{th}}}-1\right]-I_{S3}\left[e^{\frac{V+IR_{S}\left(N_{s}/N_{sh}\right)}{\eta_{3}\;N_{s}V_{th}}}-1\right]\right)-\frac{V+IR_{S}\left(N_{s}/N_{sh}\right)}{N_{s}N_{sh}R_{sh}}$$

### 2.3 Objective function

The following formula has been applied according to the root mean square error (RMSE) during the statistical assessment in this study [47,48]:

(4) $$Objective=\mathrm{Min}\left(RMSE\right)=\mathrm{Min}\left(\sqrt{\frac{1}{N_{c}}\sum_{c=1}^{N_{c}}{\left(I_{Est,c}\left(V_{\mathrm{Exp},c},x\right)-I_{\mathrm{Exp},c}\right)}^{2}}\right)$$

where *I*_*Exp,c*_ and *V*_*Exp,c*_ illustrate the measured readings of current and voltage for each record c, respectively; *N*_*c*_ displays the measured readings' number; *x* symbolizes the solution vector that can be summarized as illustrated in Equation (5) [49]:

(5) $$x=\left[I_{ph}\;R_{sh}\;R_{S}\;I_{S1}\;\eta_{1}\;I_{S2}\;\eta_{2}\;I_{S3}\;\eta_{3}\;\right]$$

## 3 Proposed improved Kepler optimization algorithm (IKOA) for PV module parameter identification

### 3.1 Standard KOA

KOA is a unique metaheuristic version inspired from Kepler's calculations for predicting the position and velocity of the planets at a specific moment [39]. In KOA, each planet's position functions as a potential solution that is arbitrarily changed with respect to the Sun, which stands for the ideal candidate solution, throughout the optimization process. Thus, a population size of *N*_*p*_ planets will be randomly given in Dim dimensions, reflecting the potential variables of an optimization problem as illustrated in Equation (6) [50]:

(6) $${\vec{X}}_{i,j}\left(0\right)={\vec{X}}_{j,low}+r_{1}\times \left({\vec{X}}_{j,up}-{\vec{X}}_{j,low}\right),i=1:N_{p};j=1:\mathrm{Dim}$$

where *r* is an integer that is generated randomly and has a range of 0–1, and *X*_*i,j*_ is a solution vector that represents each planet. Similar to other metaheuristics, the KOA is initialized randomly [51], the constraints *X*_*j,low*_ and *X*_*j,up*_ are the lower and upper limits of each control variable (*j*), accordingly. In this framework, nine unknown components have to be projected from the *I*-*V* data of PV modules [52,53].

Also, the criterion of optimal solution is determined based on the minimum objective score that is applied during the statistical evaluation in this study according to RMSE as mathematically modeled in Eq. (4). The velocity of each object is then calculated based on its position in relation to the sun. Eq. (7) provides a mathematical formulation for this motion pattern [40].

(7) $$V_{i}\left(t\right)=\left\{ \begin{array}{cc} r_{4}\times \left({\vec{X}}_{a}-{\vec{X}}_{i}\right)\times H+F\times \left(1-R_{i-norm}\left(t\right)\right)\times {\vec{U}}_{2}\times {\vec{r}}_{5}\left(r_{3}{\vec{X}}_{i,up}-{\vec{X}}_{i,low}\right) & if\;R_{i-norm}\left(t\right)>0.5\\ \rho \left(2r_{4}{\vec{X}}_{i}-{\vec{X}}_{b}\right)-\rho^{*}\left({\vec{X}}_{b}-{\vec{X}}_{a}\right)+F\times {\vec{U}}_{1}\times {\vec{r}}_{5}\times \left(1-R_{i-norm}\left(t\right)\right)\left({\vec{X}}_{i,up}-{\vec{X}}_{i,low}\right) & Else \end{array}\right.$$

Where H,ρ, and $\rho^{*}$ are mathematically defined in Equations (8), (9), (10), respectively [39].

(8) $$H={\left[\mu \left(t\right)\times \left(Ms+m_{i}\right)\left|\frac{2}{R_{i}\left(t\right)+\varepsilon }-\frac{1}{a_{i}\left(t\right)+\varepsilon }\right|\right]}^{0.5}$$

(9) $$\rho =\vec{U}\times \left(r_{3}\times \left(1-r_{4}\right)+r_{4}\right)\times H$$

(10) $$\rho^{*}=\left(1-\vec{U}\right)\times \left(r_{3}\times \left(1-r_{5}\right)+r_{5}\right)\times H$$

where *U*, *U*_*1*_, *U*_*2*_ are typically random integers chosen from the range of numbers{0,1}; The *i*th object's velocity at time *t* is designated by *V*_*i*_*(t)* with position represented by *X*_*i*_; a vector form is indicated by the symbol (→); *F* manifests a randomly chosen integer that is a member of the set −1,1; the values (*r*_*1*_, *r*_*2*_, *r*_*3*_, *r*_*4*_, and *r*_*5*_) demonstrate uniformly distributed random numbers that fall inside the range of [0,1]; The universal gravitational constant is denoted by *μ*(*t*); *ε* illustrates small value to prevent division by zero mistakes; *Ms* and *m*_*i*_ clarify the masses of *X*_*s*_ and *X*_*i*_, respectively; *X*_*a*_ and *X*_*b*_ are randomly selected options from the total population; *R*_*i*_*(t)* represents the distance among each object *X*_*i*_ and the sun *X*_*s*_ at any *t* time. According to Kepler's third rule, defined in Eq. (11), *a*_*i*_ (*t*) determines the semimajor axis of the object *i* elliptical orbit at *t* [39]:

(11) $$a_{i}\left(t\right)=r_{3}\times {\left[{T_{i}}^{2}\times \frac{\mu \left(t\right)\times \left(Ms+m_{i}\right)}{4\pi^{2}}\right]}^{\frac{1}{3}}$$

where *T*_*i*_ is the orbital interval, expressed as an absolute value generated by the normal distribution, for each *i*th item *i*. Standardizing the Euclidian distance across *X*_*i*_ and *X*_*s*_ can be seen by the notation *R*_*i−norm*_*(t)* in Equation (12) [39]:

(12) $$R_{i-norm}\left(t\right)=\left(R_{i}\left(t\right)-R_{\min}\left(t\right)\right)/\left(R_{\max}\left(t\right)-R_{\min}\left(t\right)\right)$$

Objects move momentarily closer to and then further from the Sun as they revolve around it. KOA illustrates this behavior in two fundamental steps: exploration and exploitation. KOA searches for novel places close to the best answers by looking into objects far from the sun, and it employs more precise solutions found closer to the sun to find stuff far from the sun. The positions of all objects that are far from the Sun are modified in the manner that follows the previous phases [39]:

(13) $${\vec{X}}_{i}\left(t+1\right)={\vec{X}}_{i}\left(t\right)+{\vec{V}}_{i}\left(t\right)\times F+\vec{U}\times \left(Fg_{i}\left(t\right)+\left|r\right|\right)\times \left({\vec{X}}_{s}\left(t\right)-{\vec{X}}_{i}\left(t\right)\right)$$

where *X*_*s*_ (*t*) specifies the Sun's position concerning the chosen optimal solution, *F* is used as a flag to change the search parameters, and *X*_*i*_*(t +1)* denotes the newly found location at *t*+1of for every planet *i*. Moreover, the gravitational force that attracts any planet *X*_*i*_ to the Sun *X*_*s*_ can be represented by the symbol *Fg*_*i*_ as illustrated in Equation (14) [39]:

(14) $$Fg_{i}\left(t\right)=e_{i}\times \mu \left(t\right)\times \frac{\left(\vec{Mn_{s}}\times {\vec{mn}}_{i}\right)}{{{\vec{Rn}}_{i}}^{2}+\varepsilon }+r_{4}$$

where *e*_*i*_ manifests a number among 0 and 1 that denotes the oddness of an orbiting planet, which was added to provide a stochastic quality for the original KOA; and the symbols *Mn*_*s*_ and *mn*_*i*_ represent the normalized values of *Ms* and *m*_*i*_ that designate the masses of *Xs* and *Xi*, respectively, as described in Equations (15), (16), respectively [40]:

(15) $$Mn_{s}=r_{2}\times \left(fit_{s}\left(t\right)-worst\left(t\right)\right)/\sum_{k=1}^{N_{p}}\left(fit_{k}\left(t\right)-worst\left(t\right)\right)$$

(16) $$mn_{i}=\left(fit_{i}\left(t\right)-worst\left(t\right)\right)/\sum_{k=1}^{N_{p}}\left(fit_{k}\left(t\right)-worst\left(t\right)\right)$$

Additionally, *Rn*_*i*_ denotes the normalized value of *R*_*i*_ that corresponds to the Euclidian distance as illustrated in Equation (17) [40]:

(17) $$Rn_{i}\left(t\right)={\left\|X_{s}\left(t\right)-X_{i}\left(t\right)\right\|}_{2}={\left(\sum_{j=1}^{\mathrm{Dim}}{\left(X_{s}\left(t\right)-X_{i}\left(t\right)\right)}^{2}\right)}^{\frac{1}{2}}$$

where *worst(t)* indicates the option for the best outcome with the highest fitness rating. *r*_*2*_ is an integer between 0 and 1 that is chosen at random and used to divide the combined weight of many planets. *μ*(*t*) is an expression that exponentially decreases with time (*t*) so that it controls the precision of searches. *μ*(*t*) can be defined in Equation (18) [40]:

(18) $$\mu \left(t\right)=\mu_{o}\times e^{-\gamma \times t/T_{\max}}$$

where *γ* stands for a constant, *t* characterizes the current iteration number, and *T*_max_ signifies the entire number of iterations.

KOA will prioritize improving the exploitation operation when a planet is near the sun; if the sun is far away, it will prioritize improving the exploration operator. This idea's mathematical representation can be applied in the following ways to enhance both operators [40]:

(19) $${\vec{X}}_{i}\left(t+1\right)={\vec{U}}_{1}\times {\vec{X}}_{i}\left(t\right)+\left(\frac{{\vec{X}}_{s}+{\vec{X}}_{a}\left(t\right)+{\vec{X}}_{i}\left(t\right)}{3}+\frac{1}{e^{\left(r\times \left(1+\left(a_{2}-1\right)\times r_{4}\right)\right)}}\times \left(\frac{{\vec{X}}_{i}\left(t\right)+{\vec{X}}_{s}+{\vec{X}}_{a}\left(t\right)}{3}-{\vec{X}}_{b}\left(t\right)\right)\right)\times \left(1-{\vec{U}}_{1}\right)$$

where *a*_*2*_ denotes a cyclical control parameter that progressively decreases from 2 to 1 for *T* cycles throughout the optimizing activity, as illustrated in Equation (20) [39], and *r* reflects a random integer generated using the normal distribution.

(20) $$a_{2}=-1-1\times \left(\frac{t}{T_{\max}}\right)$$

The last stage (elitism) ensures the optimal placements for the planets and the Sun through the use of an elitist approach. Eq. (21) provides an outline of this process [39]:

(21) $${\vec{X}}_{i,new}\left(t+1\right)=\left\{ \begin{array}{cc} {\vec{X}}_{i}\left(t+1\right) & if\;f\left({\vec{X}}_{i}\left(t+1\right)\right)\leq f\left({\vec{X}}_{i}\left(t\right)\right)\\ {\vec{X}}_{i}\left(t\right) & Else \end{array}\right.$$

### 3.2 Proposed IKOA

In KOA, the planet positions are determined and updated by the aforementioned KOA using two different updating methods. Firstly, the exploration method for distant objects from the sun is represented by Equation (13) whereby their positions are modified according to their anticipated velocities. Secondly, the exploitation technique for objects close to the sun is represented by Equation (19). The transfer between both procedures is carried out by means of a comparison between two arbitrary numbers {r, r1}. An equal path is generally corresponding to this transfer mechanism. As a result, at the beginning of the iteration trip, the exploitation strategy will be initiated using approximately half of the solutions for each iteration. To overcome this challenge, the standard KOA is enhanced by the addition of a Local Escaping Operator (LEO) to produce an IKOA, which leads to a better method of exploring with avoiding local optima. After every iteration, the KOA method modifies the outcomes using the proposed mathematical model as illustrated in Equation (22):

(22) $${\vec{X}}_{i,new}\left(t+1\right)=\left\{ \begin{array}{cc} {\vec{X}}_{i}\left(t+1\right)+\left(\beta_{1}{\vec{X}}_{s}-\beta_{2}{\vec{X}}_{kv}\right)\;\phi_{1}+\left({\vec{X}}_{R1}-{\vec{X}}_{R2}\right)\sigma_{1}\phi_{2}\beta_{2}/2 & if\;r_{3}<0.5\\ \left(\beta_{1}{\vec{X}}_{s}-\beta_{2}{\vec{X}}_{kv}\right)\phi_{1}+\left({\vec{X}}_{R1}-{\vec{X}}_{R2}\right)\sigma_{1}\phi_{2}\beta_{2}/2+{\vec{X}}_{s} & Else \end{array}\right.if\;r_{4}<Q_{w}$$

where *X*_*R1*_ and *X*_*R2*_ illustrate two randomly chosen solutions from the population; *ϕ*_*1*_ and *ϕ*_*2*_ denote two randomized values within the set [-1; 1] which can be reached from a uniform distribution function. The *r*_*3*_ and *r*_*4*_ indicate randomized values within [0, 1]. Additionally, the probability factor (*Q*_*w*_) will regulate the LEO triggering.

Similarly, the two randomised numbers, denoted by *β*_*1*_ and *β*_*2*_, can be calculated using Equations (23), (24):

(23) $$\beta_{1}=2\times r_{5}\times V_{o}-\left(V_{o}-1\right)$$

(24) $$\beta_{2}=r_{5}\times V_{o}-\left(V_{o}-1\right)$$

(25) $$V_{o}=\left\{ \begin{array}{cc} 0 & V_{1}>0.5\\ 1 & Else \end{array}\right.$$

where *V*_*0*_ signifies an integer from the range [0, 1] which randomly generated as illustrated in Equation (25).

Fig. 2 displays the stages for implementing the IKOA.

### 3.3 IKOA methodology for PV parameter estimation: assumptions, limitations, and uncertainties

As shown in the pervious subsection, the proposed IKOA methodology is described, and its consecutive steps are illustrated in Fig. 2 an overview of the assumptions, limitations, and uncertainties [54] associated to the methodology employed for the identification of photovoltaic (PV) module characteristics using the advanced IKOA is denoted in this section,

Firstly, several assumptions made during the course of the study.

• The validity and applicability of the 3DM for describing the electrical behavior of the PV modules under consideration.
• Constant solar irradiance and temperature conditions are considered during the parameter identification process to simplify the analysis.
• Steady-state operation of the PV modules during parameter identification is handled, neglecting transient effects.

Secondly, certain limitations inherent in the presented methodology can be summarized as follows.

• Algorithm Efficiency: While KOA offers a novel approach to parameter identification, its efficiency may be further improved to augment convergence accuracy and speed.
• Model Complexity: The complexity of the 3DM and the computational burden associated with parameter identification may limit the scalability of the proposed approach to larger PV systems.

Thirdly, there are some presence of uncertainties in the study where uncertainties in the measurement of PV module characteristics, such as current-voltage (I-V) curve data, may affect the accuracy of parameter identification results.

By recognizing these assumptions, limitations, and uncertainties, a transparent and comprehensive account of the proposed methodology and its potential implications is provided for the interpretation of the obtained results.

## 4 Simulation results and discussions

The temperature of the solar PV panel's cells has a significant impact on its performance. Consequently, various types of PV are well-suited to the specific climatic conditions found in different regions, as exemplified by the study conducted in Libya [55]. In this article, the suggested IKOA besides the standard KOA are extended for approximating PV parameters for three various modules which are STM6-40/36, Photowatt PWP201 and R.T.C France. The STM6-40/36 is the first module; it consists of 36 monocrystalline cells connected in series, each measuring 38 mm × 128 mm at 51 °C and 1000 W/m^2^ of radiation [56]. The Photowatt-PWP-201 is the second module, featuring 36 polycrystalline silicon series cells at a temperature of 45 °C and an irradiation of 1000 W/m^2^ [57]. For this module, the electrical specifications are 1.03163 A, 16.7753 V, 0.9162 A, 12.6049 V and 11.55 W for the short circuit current, open circuit voltage, maximum power point current, voltage and power, respectively. Also, the commercially available silicon R.T.C France cell is addressed which operates at sun irradiance of 1000 W/m^2^ and temperature of 33 °C. For this module, the electrical specifications are 0.7603 A, 0.5728 V, 0.6894 A, 0.4507 V and 0.3107 W for the short circuit current, open circuit voltage, maximum power point current, voltage and power, respectively.

### 4.1 Application for the first PV module

**Fig. 3:**
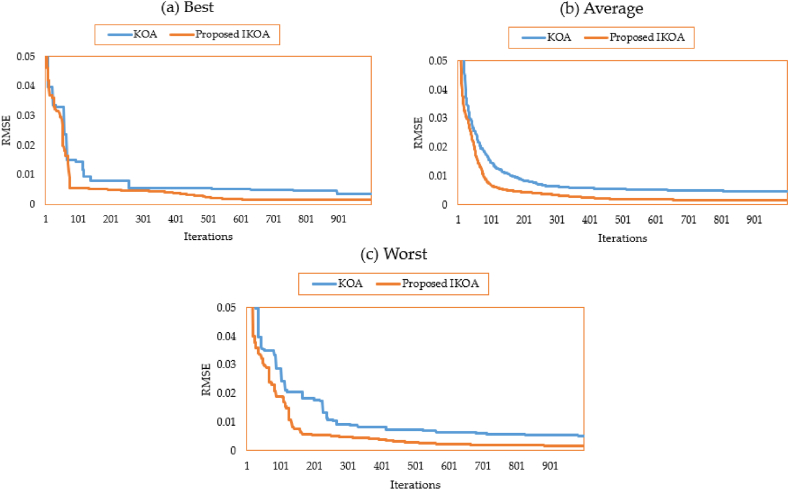
Average, best, and worst converging properties of KOA and IKOA for the first module.

The characteristics of the 3DM of the PV module of the STM6-40/36 PV module are derived for this module employing the proposed IKOA and the conventional KOA. Table 1 displays the relevant variables and findings. As illustrated, the suggested IKOA produces a result with a minimal error of 0.001689, whereas the regular KOA produces an error of 0.00354. Both approaches, the suggested IKOA and the traditional KOA, are tested for effectiveness in thirty different runs. Fig. 3(a–c) depicts their converging qualities at their best, average, and worst.

**Table 1:** Extracted parameters based on KOA and IKOA of the first module.

Item	Lower limit	Upper limit	KOA	Proposed IKOA
***I***_***Ph***_**(A)**	0.00	2.00	1.659024	1.663915
***R***_***s***_**(Ω)**	0.00	0.36	0.002692	0.007582
***R***_***sh***_**(Ω)**	0.00	1000	28.6582	17.0767
***I***_***S1***_**(A)**	0.00	50E-06	1.13E-06	2.88E-06
***η***_***1***_	1.00	2.00	2.00	1.63437
***I***_***S2***_**(A)**	0.00	50E-06	9.79E-06	7.85E-10
***η***_***2***_	1.00	2.00	1.843256	1.026534
***I***_***S3***_**(A)**	0.00	50E-06	1.04E-07	4.56E-07
***η***_***3***_	1.00	2.00	1.320949	1.797763
**RMSE**	–	–	0.00354	0.001689

In addition, with the proposed IKOA, the gathered electrical variables associated are 1.663915 A for the photo current; 0.007582 Ω and 17.0767Ω for the series resistance and shunt resistance; 2.88 μA, 78500 μA and 45.6 μA for the reverse saturation currents for D1, D2 and D3, respectively; 1.634, 1.026, and 1.797 for the ideality factors of D1, D2, and D3, respectively. As shown in Fig. 3(a–c), significant improvement is associated with the proposed IKOA from the beginning of the iterations journey. This is due to enhancing the exploration capability that is activated based on the LEO mechanism. Regarding the best converging properties, the improvement starts at the 50th iteration while this progress appears earlier in terms of the average and worst properties.

**Fig. 4:**
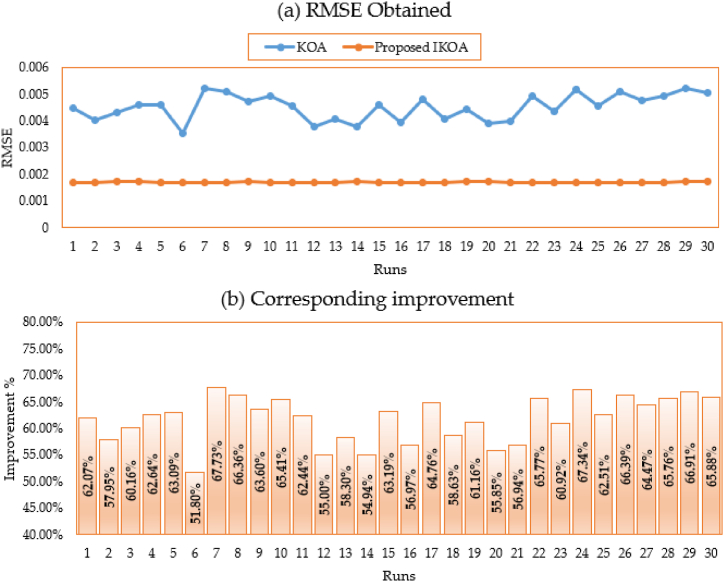
Obtained RMSE for all runs based on KOA and IKOA and the associated improvement for the first module.

In addition to that, Fig. 4(a, b) depicts the disparate obtained RMSE for all executed runs based on KOA and IKOA while the associated improvement of the proposed IKOA over KOA is displayed as well. As shown, the proposed IKOA derives greater superiority and robustness over the KOA. It shows an average improving rate of 62.27 % whereas their minimum and maximum improvement is reached to 52.28 % and 67.08 %, respectively.

**Fig. 5:**
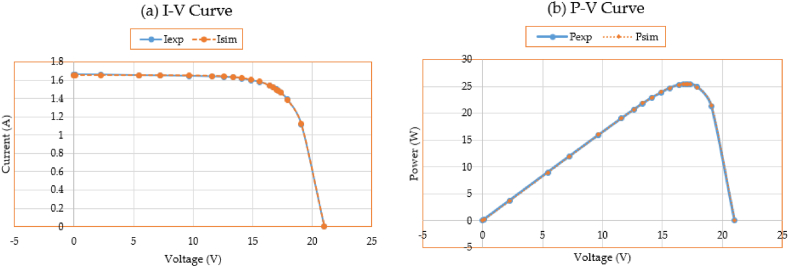
Measured and estimated data for the 3DM design of the first module.

Additionally, Fig. 5(a, b) shows the simulated performance of the P-V and I-V characteristics making use of the results of the 3DM design in comparison to the information utilized for the parameters’ estimation. When calculating the PV characteristics based on the suggested IKOA, the presented P-V and I-V curves demonstrate a great effective correlation among the observed and estimated data.

Additionally, a comparison of the suggested IKOA, KOA, and other newly established methodologies, such as the tuna swarm method (TSM) [58], teaching learning studying-based algorithm (TLSBA) [[59], [60], [61]] and African vultures optimizer (AVO) [62] and reported technique of artificial electric field optimizer (AEFO) [63], artificial hummingbird optimizer (AHO) [64], social network search (SNS) technique [65] and enhanced SNS, for this model, is explained in Table 2. This table points that the RMSE values for the Min, Mean, Max, and Standard Deviation are 1.6889E-03, 1.71E-03, 1.72E-03, and 1.06E-05, accordingly. The acquired findings show that, in comparison to other recently created competing strategies, the used IKOA expands the considerable improvement and efficacy for best 3DM description.

**Table 2:** Statistical comparisons of KOA and IKOA versus other reported methods for the first module.

Optimizer	Max (RMSE)	Mean (RMSE)	Min (RMSE)	Std (RMSE)
**SNS** [17]	3.34643E-3	2.829747E-3	2.30797E-3	3.3307E-4
**AEFO** [63]	–	–	1.7203E-3	–
**TSLBA** [64]	4.982E-3	3.4767E-3	2.2864E-3	6.4695E-4
**AVO** [64]	5.976E-3	4.5393E-3	3.5398E-3	6.4884E-4
**Enhanced SNS** [17]	2.0521E-3	1.7377E-3	1.7025E-3	3.3307E-4
**TST** [64]	6.267E-3	4.9114E-3	2.2010E-3	8.0814E-4
**AHO** [64]	2.4504E-3	1.7979E-3	1.6945E-3	1.6336E-4
**KOA**	5.24E-03	4.52E-03	3.54E-03	4.851E-04
**Proposed IKOA**	1.72E-03	1.71E-03	1.6889E-03	1.061E-05

### 4.2 Application for the second PV module

In this instance, the suggested IKOA and the traditional KOA are used to extract the 3DM parameters from the PV panel of the Photowatt PWP201 PV module. The pertinent data is included in Table 3 along with the RMSE findings. The proposed IKOA, as shown, yields a result with a minimal error of 0.0024251, whereas the conventional KOA yields an error of 0.0029458. The obtained electrical variables connected with the proposed IKOA are 1.03051 A for the photo current; 0.03372 Ω and 27.27 Ω for the series resistance and shunt resistance; 3.48 μA, 4.38E-20 A and 8.24E-19 A for the reverse saturation currents for D1, D2 and D3, respectively; 1.35, 1.7, and 1.596 for the ideality factors of D1, D2, and D3, respectively.

**Table 3:** Extracted parameters for the second module based on KOA and IKOA.

Item	Lower limit	Upper limit	KOA	Proposed IKOA
***I***_***Ph***_**(A)**	0	2	1.0320020	1.0305143
***R***_***s***_**(Ω)**	0	2	0.0341740	0.0333686
***R***_***sh***_**(Ω)**	0	2000	32.7226594	27.2772837
***I***_***S1***_**(A)**	0	50E-06	1.91E-05	3.48E-06
***η***_***1***_	1	2	1.9086835	1.3511899
***I***_***S2***_**(A)**	0	50E-06	0.00E+00	4.38E-20
***η***_***2***_	1	2	1.6618056	1.6993924
***I***_***S3***_**(A)**	0	50E-06	1.28E-06	8.24E-19
***η***_***3***_	1	2	1.2652925	1.5958645
**RMSE**	–	–	0.0029458	0.0024251

**Fig. 6:**
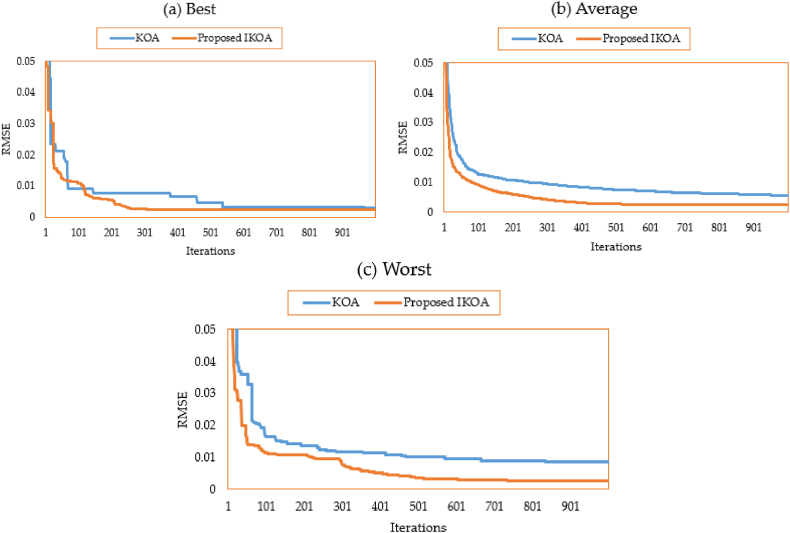
Best, average and worst converging properties of KOA and IKOA for the second module.

Both approaches, the suggested IKOA and the traditional KOA, are tested for effectiveness in thirty different runs. Fig. 6(a–c) depicts their converging qualities at their best, average, and worst.

Significant progress is linked to the suggested IKOA from the start of the iterative trip, as seen in Fig. 6(a–c). This is because the exploration capacity that is enabled through the LEO technique has been improved. The improvement begins with the best converging qualities at iteration 110, whereas it starts earlier with the average and worst properties.

**Fig. 7:**
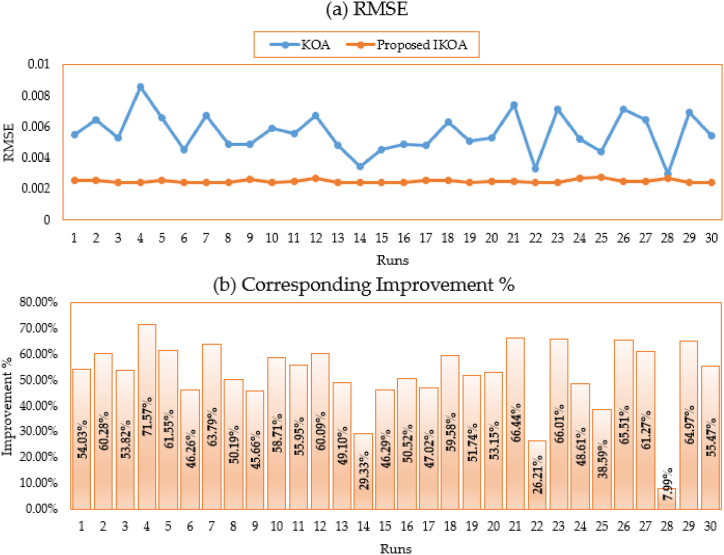
Obtained RMSE for all runs based on KOA and IKOA and the associated improvement for the second module.

Further, Fig. 7(a and b) shows the divergent achieved root mean square error (RMSE) for all runs based on KOA and IKOA, as well as the benefit of the proposed IKOA over KOA. As shown, the proposed IKOA derives greater superiority and robustness over the KOA. It shows an average improving rate of 55.1 % whereas their minimum and maximum improvement reached 17.68 % and 68.14 %, respectively. On the level of standard deviation, 92.58 % improvement is accompanied to the proposed IKOA versus the conventional KOA.

**Fig. 8:**
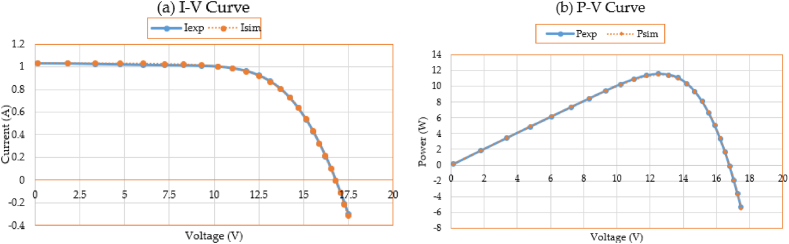
Measured and estimated data for the second module.

Moreover, Fig. 8(a, b) displays the simulated performance of the I-V and P-V characteristics making use of the results of the 3DM design in comparison to the information utilized for the parameters’ estimation. When calculating the PV characteristics based on the suggested IKOA, the presented P-V and I-V curves demonstrate a great effective correlation among the observed and estimated data.

Table 4 also displays a comparison of the recommended IKOA, KOA, and other recently developed techniques. The acquired findings show that, in comparison to other recently created strategies, the employed IKOA enhances the considerable improvement and efficacy for optimum 3DM design. It is evident that the suggested IKOA, with RMSE values of 2.4251E-03, 2.4970E-03, 2.7178E-03, and 9.5409E-05, respectively, obtains the lowest RMSE values in all indicators for the minimum, average, maximum, and standard Deviation.

**Table 4:** Statistical comparisons of KOA and IKOA versus other reported methods for the second module.

Method	Min (RMSE)	Mean (RMSE)	Max (RMSE)	Std (RMSE)
**Cuckoo Search Algorithm** [66]	3.2000E-03	–	–	–
**Sunﬂower optimization (SFO)** [43]	8.2500E-02	–	–	–
**Artificial ecosystem-based optimizer (AEO)** [67]	2.4800E-03	–	–	–
**Biogeography-based Heterogeneous Cuckoo Search (BHCS)** [68]	3.6790E-03	–	–	–
**Social network search algorithm (SNS)** [17]	2.5090E-03	3.1910E-03	5.5110E-03	2.5090E-03
**PSO** [69]	3.3925E-3	2.0808E-2	3.3742E-2	–
**KOA**	2.9458E-03	5.5609E-03	8.5294E-03	1.2862E-03
**Proposed IKOA**	2.4251E-03	2.4970E-03	2.7178E-03	9.5409E-05

### 4.3 Application for the R.T.C France PV cell

**Fig. 9:**
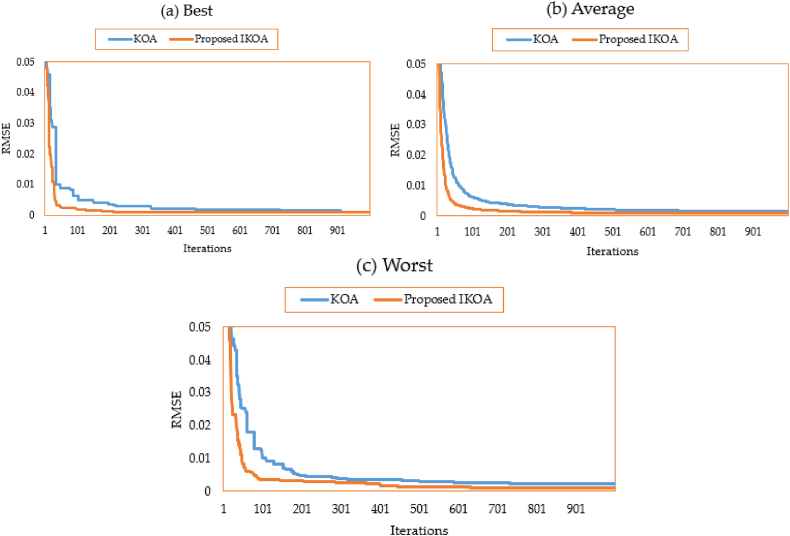
Best, average and worst converging properties of KOA and IKOA for the R.T.C France PV cell.

In this case, the parameters of 3DM of the R.T.C France PV cell are extracted using the proposed IKOA and the conventional KOA. Table 5 displays the relevant variables and findings. As illustrated, the suggested IKOA produces a result with a minimal error of 0.00098249, whereas the regular KOA produces an error of 0.00112888. Therefore, the proposed IKOA derives 12.97 % improvement compared to the KOA. Fig. 9(a–c) depicts their converging qualities at their best, average, and worst. As demonstrated, the best, average, and worst attributes significantly improve with the suggested IKOA from the beginning of the iterative trip.

**Table 5:** Extracted parameters for the R.T.C. France photovoltaic cell based on KOA and IKOA.

Item	Lower limit	Upper limit	KOA	Proposed IKOA
***I***_***Ph***_**(A)**	0.00	1.00	0.76045130	0.76078104
***R***_***s***_**(Ω)**	0.00	100.00	0.03571450	0.03675121
***R***_***sh***_**(Ω)**	0.00	0.50	58.19115098	55.56404548
***I***_***S1***_**(A)**	0.00	10E-06	0.00000029	0.00000064
***η***_***1***_	1.00	2.00	2.00000000	1.99999956
***I***_***S2***_**(A)**	0.00	10E-06	0.00000000	0.00000013
***η***_***2***_	1.00	2.00	1.35663919	1.99984559
***I***_***S3***_**(A)**	0.00	10E-06	0.00000034	0.00000022
***η***_***3***_	1.00	2.00	1.48955556	1.45003885
**RMSE**	–	–	0.00112888	0.00098249

**Fig. 10:**
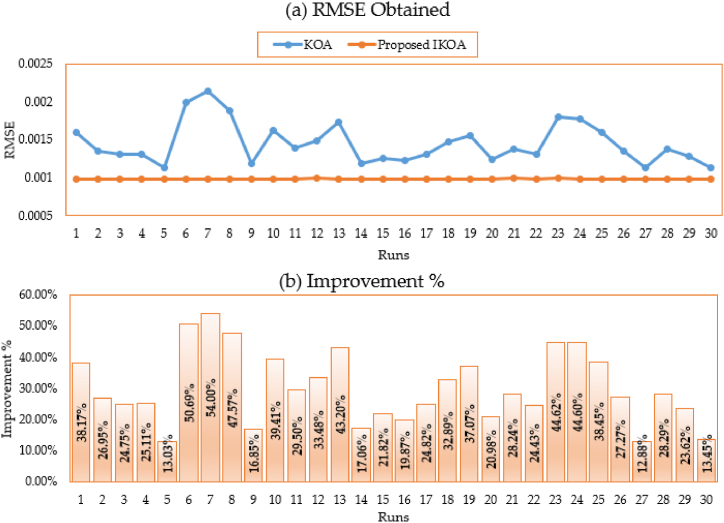
Obtained RMSE for all runs based on KOA and IKOA and the associated improvement for the R.T.C France PV cell.

In addition to that, Fig. 10(a, b) depicts the disparate obtained RMSE for all executed runs based on KOA and IKOA while the associated improvement of the proposed IKOA over KOA is displayed as well. As shown, the proposed IKOA derives greater superiority and robustness over the KOA. It shows an average improving rate of 32.12 % whereas their maximum improvement reached 53.47 %, respectively. On the level of standard deviation, 98.87 % improvement is accompanied to the proposed IKOA versus the conventional KOA.

**Fig. 11:**
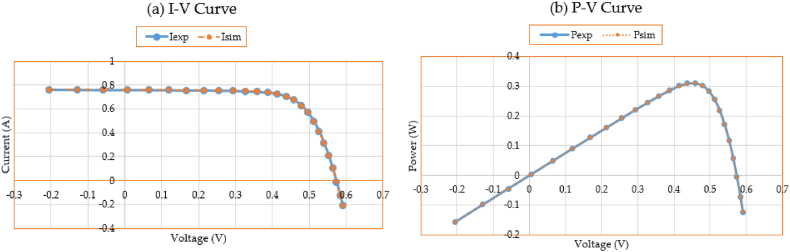
Measured and estimated data for the R.T.C France PV cell.

Furthermore, Fig. 11 (a, b) compares the data used for parameter estimate to the simulated performance of the I-V and P-V characteristics using the outcomes of the 3DM design. The depicted I-V and P-V curves show a strong effective correlation among the calculated and observed data when computing the PV characteristics according to the prescribed IKOA.

Table 6 also displays a comparison of the recommended IKOA, KOA, and other recently developed techniques. The acquired findings show that, in comparison to other recently created strategies, the employed IKOA enhances the considerable improvement and efficacy for optimum 3DM design. It is evident that the suggested IKOA, with RMSE values of 9.82490E-04, 9.85769E-04, 9.97427E-04 and 3.02730E-06, respectively, obtains the lowest RMSE values in all indicators for the Min, Mean, Max, and Standard Deviation.

**Table 6:** Statistical comparisons of KOA and IKOA versus other reported methods for the R.T.C France PV cell.

Method	Min (RMSE)	Mean (RMSE)	Max (RMSE)	Std (RMSE)
**Growth optimizer** [45]	9.8393E-04	9.9793E-04	1.002E-03	6.4133E-06
**Energy valley optimizer (EVO)** [45]	1.083E-03	2.3850E-03	5.361E-03	1.644E-03
**Five Phases Algorithm (FPA)** [45]	1.1083E-03	1.2651E-03	1.431E-03	1.127E-04
**Hazelnut tree search (HTS) algorithm** [45]	1.186E-03	1.556E-03	1.988E-03	2.81E-04
**TLBO** [70]	1.52057E-03	–	–	–
**Sine cosine approach** [71]	9.86863E-04	–	–	–
**Artificial bee colony (ABC)** [72]	1.28482E-03	–	–	–
**Teaching–learning–based ABC** [73]	1.50482E-03	–	–	–
**Generalized oppositional TLBO** [37]	4.43212E-03	–	–	–
**Comprehensive learning PSO** [74]	1.3991E-03	–	–	–
**Flower pollination algorithm** [75]	1.934336E-03	–	–	–
**Cat swarm algorithm** [76]	1.22E-03	–	–	–
**KOA**	1.12888E-03	1.45218E-03	2.14378E-03	2.67700E-04
**Proposed IKOA**	9.82490E-04	9.85769E-04	9.97427E-04	3.02730E-06

### 4.4 Discussion on the cost effectiveness of KOA versus proposed IKOA

Evaluating the cost-effectiveness of optimization algorithms involves considering various factors to determine their efficiency and practicality in achieving desired outcomes while minimizing resource expenditure. Firstly, the computational cost, including time and memory requirements, is a crucial aspect. Secondly, the quality of solutions obtained relative to the computational cost is essential. The comparative approaches were executed using the MATLAB environment (MATLAB2017b) on a personal computer featuring an Intel(R) Core (TM) i7-3632QM CPU operating at 2.20 GHz and 8 GB of RAM. Table 7 displays a comparative indexes between the standard KOA and the proposed IKOA in terms of elapsed time (sec), memory used by MATLAB (MB) and average RMSE score. As shown, while the proposed IKOA shows slightly slower than the standard KOA, it derives lower memory requirements with 1495, 1492 and 1480 MB than KOA with counterparts of 1496, 1498 and 1494 MB. In terms of solution quality, the proposed IKOA shows an average improvement of more than 50 % for the three considered tested PV systems.

**Table 7:** KOA versus proposed IKOA: Elapsed time (sec), memory used by MATLAB (MB) and solution quality.

Tested PV System	Indexes	KOA	Proposed IKOA
STM6-40/36	Elapsed time (sec)	42.37	44.7
	Memory used by MATLAB (MB)	1496	1495
	Average RMSE score	4.52E-03	1.71E-03
Photowatt-PWP-201	Elapsed time (sec)	41.94	45.37
	Memory used by MATLAB (MB)	1498	1492
	Average RMSE score	5.5609E-03	2.4970E-03
R.T.C France cell	Elapsed time (sec)	41.93	44.9
	Memory used by MATLAB (MB)	1494	1480
	Average RMSE score	1.45218E-03	9.85769E-04

In order to further compare the proposed method and model formulation in this study with other recent studied of [[77], [78], [79]]. In those studies, different effective optimizers were adopted including hybridized rat swarm optimization and pattern search [77], Modified salp swarm [78] and improved tunicate swarm optimization [79]. According to the model formulation, those studies were utilized for the 1DM, and the 2DM while the presented study considers more complex model of the 3DM formulation which has two additional designed variables to be optimized. According to the PV systems under testing, those studies were applied for two PV systems of the R.T.C France cell and the Photowatt-PWP-201 while the presented study extends the application to additional STM6-40/36 PV system. For further comparisons, the proposed IKOA is applied for the same circumstances for the 1DM and 2DM for the R.T.C France cell and the Photowatt-PWP-201 in comparison to Refs. [[77], [78], [79]] as illustrated in Table 8 and the table displays the statistical metrics. As shown, the proposed IKOA shows superior performance compared to them especially in achieving a trivial standard deviations of 2.82E-17, 2.26E-07 and 3.08E-17, respectively while the nearest counterpart value remains very far with 3.01E−07, 1.45E−06 and 1.38E−05, respectively based on the hybridized rat swarm optimization and pattern search [77].

**Table 8:** Comparative assessment of the proposed IKOA versus other reported studies.

	Algorithms	Best	Mean	Worst	Std
R.T.C France cell (1DM)	Rat swarm optimizer and pattern search [77]	9.86E−04	9.865E−04	9.87E−04	3.01E−07
	Modified salp swarm [78]	9.86E−04	9.865E−04	9.87E−04	3.01E−07
	Improved tunicate swarm optimizer [79]	9.86E−04	9.89E−04	8.07E−04	3.01E−07
	Proposed IKOA	9.86E−04	9.86E−04	9.86E−04	2.82E-17
R.T.C France cell (2DM)	Rat swarm optimizer and pattern search [77]	9.82E−04	9.84E−04	9.87E−04	1.45E−06
	Modified salp swarm [78]	9.83E−04	9.94E−04	9.99E−04	1.49E−06
	Improved tunicate swarm optimizer [79]	9.82E−04	9.9E−04	8.34E−03	1.45E−06
	Proposed IKOA	9.82E−04	9.847E−04	9.86E−04	2.26E-07
Photowatt-PWP-201 (1DM)	Rat swarm optimizer and pattern search [77]	2.42E−03	2.43E−03	2.50E−03	1.38E−05
	Modified salp swarm [78]	2.42E−03	2.54E−03	2.78E−03	1.75E−05
	Improved tunicate swarm optimizer [79]	2.42E−03	2.43E−03	2.5E−03	1.38E−05
	Proposed IKOA	2.42E−03	2.42E−03	2.42E−03	3.08E-17

### 4.5 Non-parametric tests for IKOA and KOA for the three PV systems under investigation

In order to affirm the significancy of the proposed IKOA, the investigation of non-parametric tests adds to the scope of the inquiry. For this purpose, the STM6-40/36, Photowatt PWP201 and R.T.C France are considered. The *t*-test and the Wilcoxon Signrank test are used as two-sample tests to compare the proposed IKOA and the conventional KOA for the three PV systems that are the subject of the inquiry. Table 9 lists the MATLAB results for both testing. As can be seen, with the STM6-40/36, Photowatt PWP201, and R.T.C. France, the h-value is always unity and the p-value is always a very small number, recording 4.4324E-24, 1.7125E-13, and 1.7316E-10, respectively. If the null hypothesis were correct, there would be a very small chance of seeing such extreme results, as these trivial values show. Furthermore, higher differences between the two methods under comparison are indicated by larger absolute values of t-statistic measurements.

**Table 9:** IKOA vs KOA non-parametric tests: results of *t*-test and Wilcoxon Signrank.

		STM6-40/36	Photowatt PWP201	R.T.C France
*t*-test	h	1	1	1
	p	4.4324E-24	1.7125E-13	1.7316E-10
	ci	0.0026	0.0026	0.3668E-3
		0.0030	0.0036	0.5660E-3
	tstat	31.7545	12.8425	9.5799
	df	29	29	29
	sd	4.8545E-4	0.0013	2.6667E-4
Wilcoxon Signrank test	h	1	1	1
	p	3.7896E-6	2.5631E-6	1.7344E-6

## 5 Conclusion

This paper introduced a novel advanced improved Kepler optimization algorithm (IKOA) for optimal identification of photovoltaic (PV) module characteristics in solar systems It is adopted and assessed for extracting the nine parameters of the PV triple diode model (3DM) considering three different practical PV modules. At any given time, to forecast the location and motion of planets, it replicates Kepler's laws of planetary motion. The enhanced procedure of searching with avoiding local optima was made possible by the suggested IKOA's incorporation of an upgraded Local Escaping Operator (LEO) mechanism. The exploitation method, based on this mechanism, will commence activation at the beginning of the iteration trip with roughly 50 % of the answers for each iteration. The applications are demonstrated on the STM6-40/36, Photowatt PWP201 and R.T.C France. The proposed IKOA showed great enhancement versus the standard KOA to increase the precision in identifying the PV parameters regarding the 3DM representation. Higher quality and coincidence among the experimental and simulated data of the P-V and I-V curves for the three PV modules under consideration go along with the suggested IKOA. Furthermore, the suggested IKOA asserts significant superiority and robustness over previously reported results. Furthermore, the enhanced precision and efficiency achieved through IKOA are highlighted compared to conventional methods. On the other side, the need for further exploration into the scalability and applicability of IKOA is required across diverse environmental conditions. Finally, possible future works are suggested, including extending IKOA's capabilities to incorporate additional environmental factors, refining the algorithm's performance through enhanced parameter tuning strategies, and exploring its integration of PV system in power systems.

## CRediT authorship contribution statement

**Ghareeb Moustafa:** Visualization, Supervision, Methodology, Formal analysis. **Hashim Alnami:** Writing – review & editing, Visualization, Resources, Investigation, Funding acquisition. **Ahmed R. Ginidi:** Writing – review & editing, Writing – original draft, Validation, Methodology, Formal analysis. **Abdullah M. Shaheen:** Writing – original draft, Software, Methodology, Data curation, Conceptualization.

## Data and code availability statement

Data included in article/supp. material/referenced in the article is available.

We agree that our study was deposited into a publicly available repository.

## Declaration of competing interest

The authors declare that they have no known competing financial interests or personal relationships that could have appeared to influence the work reported in this paper.

## Figures and Tables

**Table A.1:** Application of metaheuristic methods on solar cell.

Technique	PV Type	PV Model	Advantage	Disadvantage
Self-Adaptive Ensemble-incorporated with Differential Evolution (SEDE) [80]	• RTC France Si cell • PhotoWatt-PWP201	1DM 2DM	High robustness and accuracy	High computation time
Particle Swarm Optimization (PSO) [69]	Not specified	1DM 2DM	• Ease of implementation. • High accuracy level in the solution.	Stuck in local minima
Performance-Guided JAYA (PGJAYA) [81]	• RTC France Si cell • PhotoWatt-PWP201	1DM 2DM	Insufficient reliability	Acceptable accuracy
Salp Swarm Algorithm [82]	TITAN-12-50	2DM	Low computational time	• Caught by local minimums. • Convergence occurs first in the procedure.
War Strategy Optimization (WSO) [13,83]	• Photo watt • RTC France cell • PWP 201, • STM6-40/36 PV module	1DM 2DM	• New technique for PV cells parameter extraction. • Low CPU time	Insufficient robustness
Improved JAYA (IJAYA) [84]	RTC France Si cell	1DM 2DM	• A simpler and more effective technique. • Robustness and Convergence are high	• Inaccurate solution • Caught by local minima
Improved Heap-Based Algorithm (IHBA) [85]	• SQ_150 PV module • PVM 752 GaAs	1DM 2DM 3DM	• Robustness and Convergence are high. • Ease of implementation	• Long time is required to compute
Rao [86]	• PhotoWatt-PWP201 • RTC France Si cell	1DM 2DM	• The high ability to explore. • Ease of implementation	• Commercial modules tests haven't been conducted. • Caught by local minima.
Improved Mosth-Flame Optimization (IMFO) [87]	• CS6P-240P module • Q6-1380 solar cell	1DM 2DM	• It is simpler. • Convergence speed is high	• Commercial modules tests haven't been conducted. • Long time is required to compute
Modified Artificial Bee Colony (MABC) [88]	RTC France Si cell	1DM 2DM	• Insensitive to noise • High robustness and accuracy	• Parameters are necessary to be repeatedly modified. • Long time is required to compute • Achieving convergence early
Triple-Phase Teachinglearning- Based Optimization (TPTLBO) [89]	• RTC France Si cell	1DM 2DM	• Fewer control parameters • Ease of implementation • Fast convergence	• Uncertainty about the solution • High computational costs
Shuffled Frog Leaping Algorithm (SFLA) [90]	• MSX-60 • KC200GT	1DM	• Fast convergence	• Not accurate • Myriads of control parameters
Artificial Hummingbird Optimizer [91]	• STM6-40/36 • KC200GT • PWP 201	1DM 2DM	• Fewer control parameters • Fast convergence	• Long time is required to compute • .
Improved Chaotic Whale Optimization (ICWO) [92]	KC200GT	1DM, 2DM	• A lower computation cost • Ease of implementation	• Trapped inside local minimums • Inability to explore • Convergence happens first in the procedure.
Coyote Optimization Algorithm (COA) [93]	• RTC France Si cell, • PhotoWatt-PWP201, • SM55 • KC200GT, • ST40	1DM 2DM	• High convergence speed • The solution quality is high	• Convergence occurs first in the procedure. • Insufficient ability to exploit
Hybrid optimizer of Grey Wolf and Cuckoo Search (GW-CS) [94]	KC200GT	1DM	• Robust design • The solution accuracy is high. • Low possibility of local minimum trapping	Slow rate of convergence speed
Improved Equilibrium Optimizer (IEO) [95]	• RTC France Si cell • PhotoWatt-PWP201 • ST40 • SM55	1DM 2DM	• Capability to exploit and explore. • High level of accuracy	High computational costs
Orthogonally Adapted Harris Hawks Technique (OAHHT) [96]	• RTC France Si cell, • PhotoWatt-PWP201 • ST40, • SM55 • PVM 752 GaAs	1DM 2DM	• Low possibility of local minimum trapping • High convergence rates • High-quality solutions	Not specified
Marine Predators Algorithm (MPA) [97]	• MSX-60 • KC200GT	2DM	• High exploratory skills • The solution accuracy is high.	• Stuck in local minima • Convergence occurs first in the procedure.
Hunter-Prey-Based Optimization [98]	• STM6-40/36 module • RTC France cell	1DM 2DM 3DM	• Excellent degree of accuracy, • High convergence rates	Not specified
New Stochastic Slime Mould Algorithm (SMA) [99]	• RTC France Si cell • PhotoWatt-PWP201	1DM 2DM	• Capability to exploit and explore, • Excellent scale of accuracy	Long time is required to compute

## Appendix

Table A.1 illustrates several published applications of metaheuristic methods on solar cell.
